# Pain pathophysiology and pharmacology of cattle: how improved understanding can enhance pain prevention, mitigation, and welfare

**DOI:** 10.3389/fpain.2024.1396992

**Published:** 2024-08-27

**Authors:** Abigale H. Zoltick, Sabine Mann, Johann F. Coetzee

**Affiliations:** ^1^ Department of Clinical Studies, University of Pennsylvania School of Veterinary Medicine, Kennett Square, PA, United States; ^2^ Department of Population Medicine and Diagnostic Sciences, Cornell University College of Veterinary Medicine, Ithaca, NY, United States; ^3^ Department of Anatomy and Physiology, Kansas State University, Manhattan, KS, United States

**Keywords:** analgesia, pain, cow, cattle, animal welfare, pain management

## Abstract

Globally, humans rely on cattle for food production; however, there is rising societal concern surrounding the welfare of farm animals. From a young age, cattle raised for dairy and beef production experience pain caused by routine management procedures and common disease conditions. The fundamental mechanisms, nociceptive pathways, and central nervous system structures required for pain perception are highly conserved among mammalian species. However, there are limitations to a comparative approach to pain assessment due to interspecies differences in the expression of pain. The stoicism of prey species may impede pain identification and lead to the assumption that cattle lack pain sensitivity. This highlights the importance of establishing validated bovine-specific indicators of pain—a prerequisite for evidence-based pain assessment and mitigation. Our first objective is to provide an overview of pain pathophysiology to illustrate the importance of targeted analgesia in livestock medicine and the negative welfare outcomes associated with unmitigated pain. This is followed by a review of available analgesics, the regulations governing their use, and barriers to implementation of on-farm pain management. We then investigate the current research undertaken to evaluate the pain response in cattle—a critical aspect of the drug approval process. With an emphasis on emerging research in animal cognition and pain pathology, we conclude by discussing the significant influence that pain has on cattle welfare and areas where further research and modified practices are indicated.

## 1 Introduction

Cows, like humans, live in a sensory environment. Sensory modalities include temperature, pressure, chemicals, light, sound, and movement. Pain, however, is not a sensory modality; it is an affective state that represents a “subjective cerebral response” (1). What we observe in animals—withdrawal, vocalization, inappetence—is the product of nociception, or the neural processing of a noxious stimulus that is transduced from a chemical signal into an action potential (2). Without spoken language to convey the emotional experience occurring within the higher centers of the brain, any reaction to a noxious stimulus must be *interpreted* as pain (1). Consequently, much of what we understand about the physiology of pain arises from the experience of human subjects. An anthropomorphic approach to pain management in veterinary medicine, however, is misleading due to interspecies differences in the expression of pain. It is therefore imperative to develop a targeted approach to pain detection that acknowledges the unique physiology, behavioral patterns, and evolutionary history of cattle.

Molony and Kent (1997) define pain as “*an aversive sensory and emotional experience representing an awareness by the animal of damage or threat to the integrity of its tissues. It changes the animal's physiology and behaviour to reduce or avoid the damage […] and to promote recovery*” (3). Although cows cannot communicate the experience of pain through spoken language, these alterations in physiology and behavior facilitate pain detection (4)—a prerequisite for evidence-based pain assessment and mitigation. Causes of pain in cattle raised for dairy and beef include routine management procedures, such as disbudding (5), castration (6), and branding (7), as well as common disease conditions. Studies demonstrate that pain is associated with lameness (8), mastitis (9), gastrointestinal (10) and respiratory disease (11), and conditions surrounding calving, such as dystocia (12) and metritis (13). Although painful conditions are common in cattle, there is a scarcity of validated bovine-specific indicators of pain. This impedes approval of drugs labelled for pain control by the responsible agencies, such as the Food and Drug Administration (FDA) in the United States and contributes to inconsistent and inadequate use of analgesia in cattle.

Our first objective is to provide an overview of pain pathophysiology to illustrate the importance of targeted analgesia in livestock medicine and the negative welfare outcomes associated with unmitigated pain. This is followed by a review of available analgesics, regulations specific to the U.S. that govern their use, and reasons for unsatisfactory implementation of on-farm pain management. We then investigate the current research undertaken to evaluate the pain response in cattle—a critical aspect of the drug approval process. With an emphasis on emerging research in animal cognition and pain pathology, we conclude by discussing the significant influence that pain has on cattle welfare and areas where further research and modified practices are indicated. Although conditions causing pain in cattle are common and widespread, there are minimal established recommendations to guide on-farm pain management protocols, especially for painful disease conditions. Furthermore, the administration of analgesics to safeguard cattle welfare remains voluntary in the U.S., as there are no state or federal regulations enforcing their use. To secure the welfare of cattle under our care, we must prioritize on-farm pain prevention and mitigation through modified management practices and preemptive and multimodal approaches to analgesic intervention.

## 2 Overview of the physiology and pathophysiology of pain in cattle

### 2.1 Nociception and pain perception

Nociception—from the Latin “nocere,” or “to harm”—is not exclusive to humans or mammals in general (14, 15). Nociceptors are found in non-mammalian vertebrates including birds, reptiles, amphibians, and fish, as well as in invertebrates, such as nematodes, leeches, and fruit flies (16). Nociceptors are high-threshold sensory receptors that preferentially respond to mechanical, thermal, or chemical noxious stimuli that may result in tissue damage (17). These sensory receptors have free nerve endings and can be found in the skin, muscles, joints, viscera, and vasculature (18). There are two types of nociceptor axons: A*δ* and C fibers. A*δ* fibers are myelinated and rapidly conduct localizable pain often described as “sharp,” signaling potential tissue damage (17, 19). C fibers are non-myelinated and thus conduct signals more slowly. They are responsible for the perception of non-localizable pain that has been described by humans as dull, throbbing, aching (17), or burning (20). This type of pain can lead to depression and withdrawal, behavioral responses that may encourage healing (17). In healthy, undisturbed tissues, these neurons are quiescent; noxious stimuli must exceed the stimulus receptor threshold for activation to occur (18). Nociception thus serves as an evolutionarily valuable alarm system, alerting an organism to potential tissue damage.

**Figure 1:**
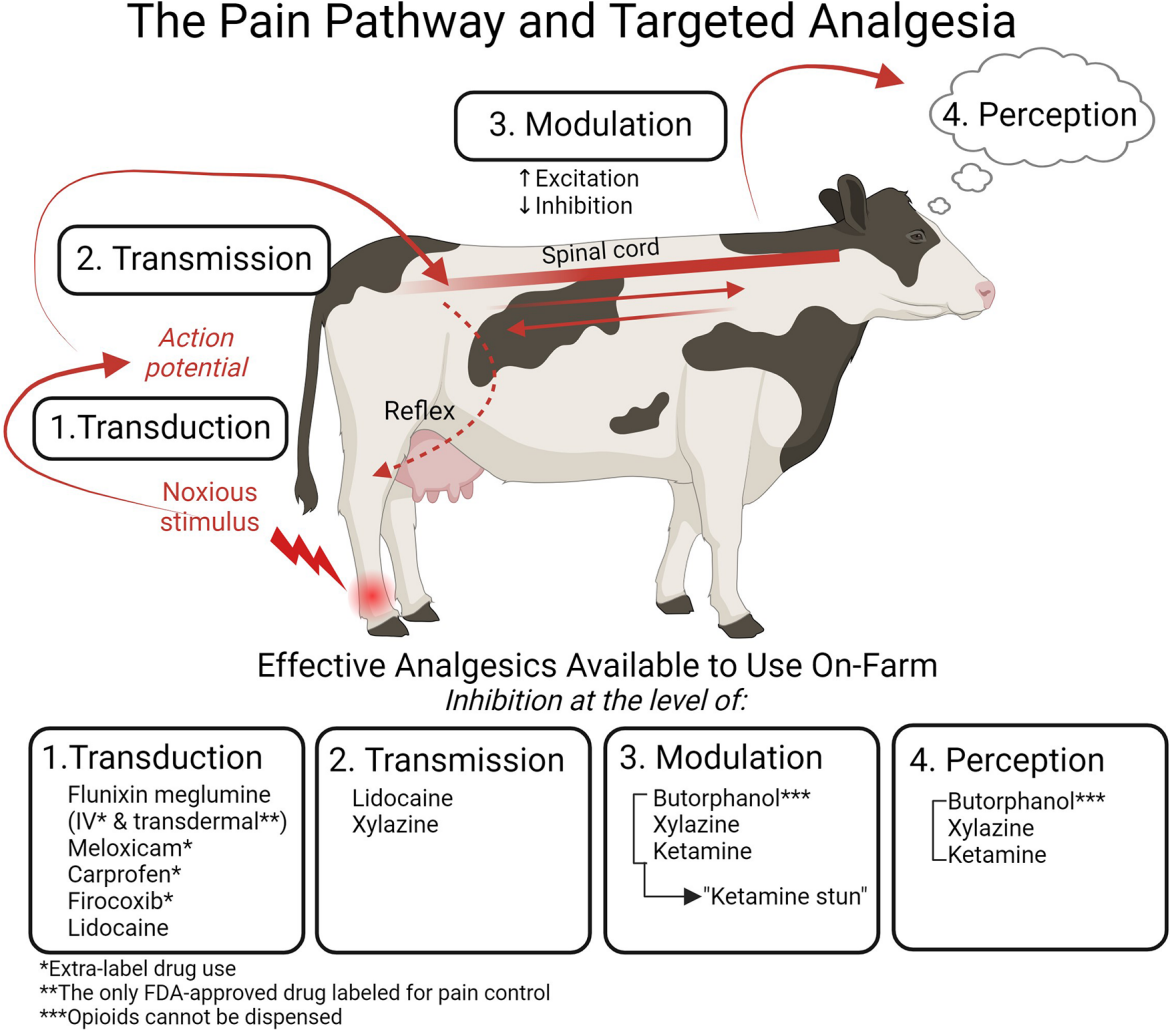
The four processes of nociception targeted by analgesia and the specific drugs available for on-farm use in cattle. Transduction (1) occurs when a noxious stimulus is converted into an action potential. This action potential then travels from the periphery to the central nervous system in the process of transmission (2). Modulation (3) results in enhancement or suppression of the nociceptive signal. Pain is experienced when the signal is perceived (4) in the higher centers of the brain. The nociceptive signal can also result in an immediate reflex response. Analgesics can target each of these processes: the NSAIDs flunixin meglumine, meloxicam, carprofen, and firocoxib inhibit transduction; the local anesthetic lidocaine inhibits transduction and transmission; the *α*_2_ agonist xylazine inhibits transmission and, when combined with the NMDA receptor antagonist ketamine and the opioid butorphanol, affects modulation and perception to inhibit pain. Flunixin meglumine transdermal is the only FDA-approved drug for pain control in cattle, and it is labelled only for foot rot; all other NSAIDs must be used in an extra-label manner. Opioids cannot be dispensed on-farm. Figure adapted from (21). Created with BioRender.com.

It is important to acknowledge the complexity surrounding nociception and pain. Nociception will not always result in pain, and pain will not always arise from nociception. To connect nociception with the experience of pain, four processes must be considered: transduction, transmission, modulation, and perception. Recognizing these disparate and interconnected processes is critical to understanding pain and targeted analgesia (Figure 1). Transduction occurs when signaling molecules, such as cytokines, bradykinin, and prostaglandins released in response to a noxious stimulus, are converted into an action potential capable of travelling to the central nervous system (18). Transmission follows when this action potential generated during transduction travels to the spinal cord via the dorsal nerve roots. It then synapses within the dorsal horn or may be propagated up the spinal cord via the ascending pathway (17). Pain modulation is a process whereby pain transmission is suppressed or heightened via inhibitory and excitatory mechanisms in both the peripheral and central nervous systems (18).

To experience pain, nociceptive information must lastly be transmitted to higher centers of the brain where the signal is processed, integrated, and recognized (20). In the brainstem, the reticular activating system (RAS) integrates signals received and projects to the thalamus and limbic system, resulting in both behavioral and autonomic pain responses (20). The thalamus projects information to the cerebrum's somatosensory cortex, which communicates with other regions of the brain including the limbic system (20). The cerebral cortex is fundamental to the experience of pain, as it is where conscious perception occurs (20). The electroencephalogram (EEG) detects cortical electrical activity, and electroencephalographic spectral analysis has been used to study pain perception in humans (22, 23) with similar EEG signal variations reported in cattle undergoing painful procedures, such as dehorning (24) and castration (25–28). EEG signal changes in response to pain include “desynchronization,” a phenomenon characterized by an increase in high-frequency activity and a reduction in low-frequency activity (29). These EEG findings signify nociceptive signal processing within the higher centers of the brain, providing evidence of pain perception in cattle.

Pain perception may be modified via cortical communication with subcortical regions, such as the thalamic and limbic systems (30). The amygdala, for example, is involved in mediating the emotional-affective aspects of pain (31). Mammalian perceived pain therefore encompasses not only sensory-discriminatory and evaluative components, but also affective state (20). Attentional and emotional states can modify pain processing, enhancing or diminishing pain perception (32). Studies in humans demonstrate diminished pain perception with distraction, positive expectation, and “positive mood manipulations,” such as playing music for a postoperative patient (32). The influence of all three of these positive contextual elements was demonstrated in a study conducted by Lomb et al. (33) in which researchers used positive reinforcement training and counterconditioning to reduce heifer reactivity to the administration of a subcutaneous injection. Heifers entered a headlock that was either closed (“habituation” heifers) or remained open (“agency” heifers) and received grain upon entrance. This food reward was then paired with gradual exposure to a sham injection. “Naïve” heifers received no treatment area exposure. “Agency” heifers demonstrated reduced approach latency compared to “habituation” and “naïve” heifers and reduced injection reactivity compared to “habituation heifers.” The researchers posited that distraction, positive expectation, and agency attenuated the fear and pain response in study subjects (33). Ede et al. (34) determined that calves receiving an intramuscular injection paired with a milk reward (1 L, 500 mL, 250 mL, and 0 mL) did not demonstrate approach latency when compared to controls until the milk reward was reduced to 250 mL, similarly suggesting that cattle experience pain attenuation in the presence of a positive stimulus.

The principal categories of pain include acute nociceptive, inflammatory, and neuropathic pain (35). Although all categories involve nociceptor activation, they have different pain-generating etiologies (36). Nociceptive pain describes the pain resulting from contact with a noxious stimulus (35); it is typically acute and dissipates upon removal of the inciting cause (36). Inflammatory pain is associated with healing and results from the release of inflammatory mediators following tissue damage that can lower the nociceptor stimulus threshold, enhancing its activation (1). It typically leads to swelling, redness, heat, and loss of function of the affected structure. Lastly, neuropathic pain occurs due to structural damage to neural components responsible for communicating nociceptive information to the central nervous system (CNS) (35). These three categories of pain are not mutually exclusive. As an example, lameness in cattle may derive from acute hoof trauma, resulting in nociceptive pain. Removal of the injurious stimulus may result in pain attenuation and healing; however, a breach in the tissues of the hoof can lead to infection, resulting in inflammatory pain. If the infection is untreated and the pain is unabated, nociceptive sensitivity may be altered, resulting in chronic, neuropathic pain.

Understanding pain etiology and the pain pathway is critical to implementing a targeted and comprehensive analgesic approach. By targeting different levels of the pathway with pharmaceutical agents, we can prevent pain and maximize analgesic efficacy (Figure 1). This preemptive and multimodal analgesic approach can protect an animal from the aversive emotional experience associated with both acute and protracted pain, as well as prevent the development of maladaptive pain—a pathological pain state that significantly impairs animal welfare.

### 2.2 Central sensitization and maladaptive pain

Adaptive pain, also referred to as acute pain, is protective; it serves to prevent sustained tissue damage by causing physiologic, neuroendocrine, and behavioral changes that promote healing (37). Conversely, maladaptive pain describes a pathological pain state in which pain persists after removal of the inciting cause and results from tissue or nerve damage (37). This pain is considered maladaptive, or “dysfunctional,” because it neither provides protection nor fosters healing (38).

**Figure 2:**
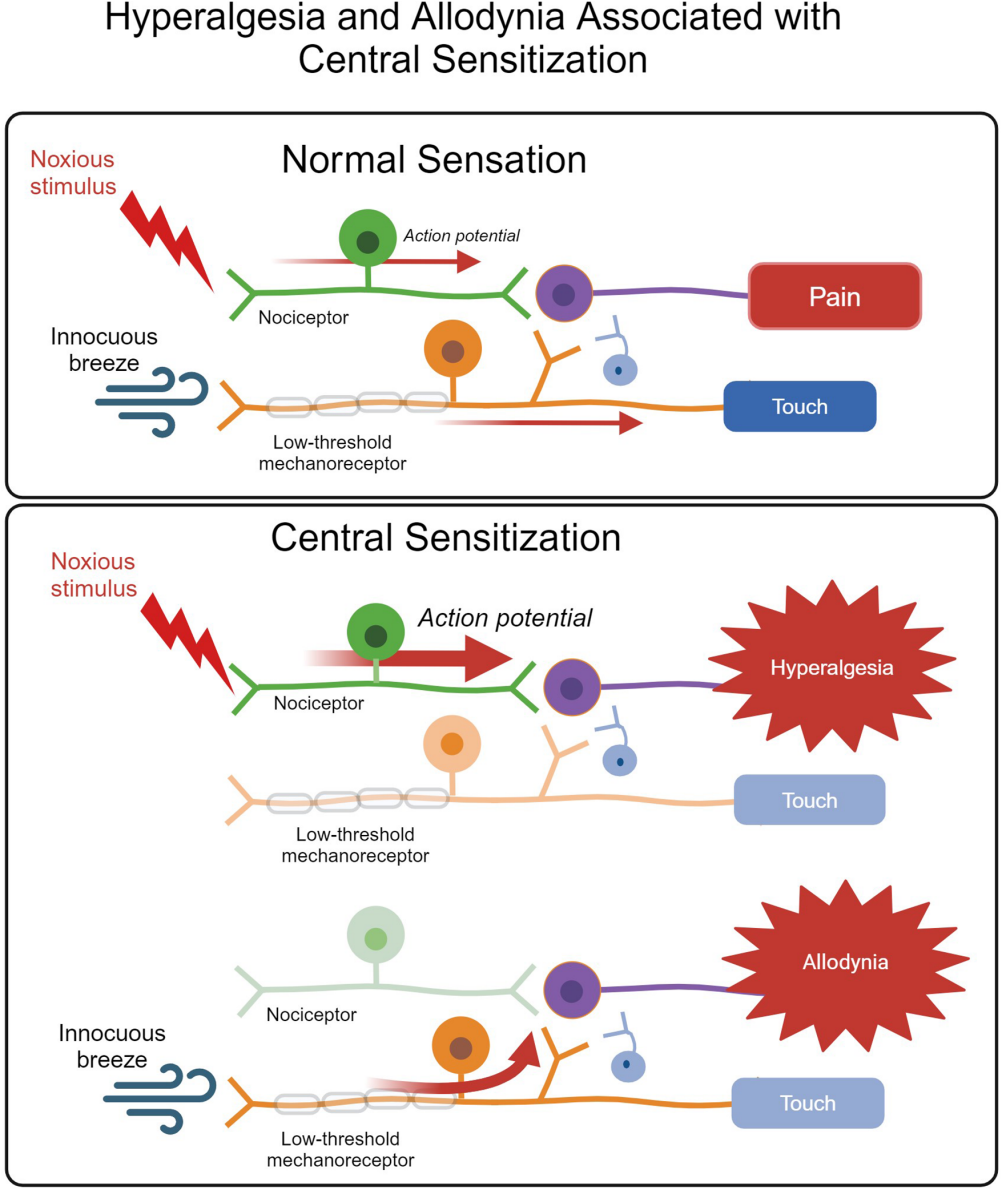
The normal somatosensory pathway for pain and touch versus heightened and abnormal activation of the pain pathway associated with central sensitization resulting in hyperalgesia and allodynia. In an animal experiencing normal sensation, noxious stimuli activate nociceptors, leading to pain. Innocuous stimuli are detected by low-threshold mechanoreceptors, which do not communicate with the pain pathway and result in touch sensation. The pathways do not communicate. Hyperalgesia describes a heightened response to nociceptive input, whereas allodynia occurs when an innocuous stimulus results in pain due to low-threshold mechanoreceptor activation of pain pathways. Figure adapted from (40). Created with BioRender.com.

Following tissue injury, neurotransmitters and inflammatory mediators, such as bradykinin and prostaglandins, surround nociceptor terminals, lowering the nociceptor's activating threshold in a process known as peripheral sensitization (1, 18). Growth factors and inflammatory cytokines and chemokines, such as tumor necrosis factor (TNF) and interleukin-1β (IL-1β), may also alter nociceptor properties via enhanced nerve terminal sensitization and nociceptor gene regulation (39). This can lead to hyperalgesia or allodynia, heightened pain sensitivity states in which mildly noxious or innocuous stimulation, respectively, results in nociceptor activation (18) (Figure 2).

In the non-diseased state, peripheral sensitization prevents an animal from further aggravating an injured area and will resolve with analgesia and as injured tissues heal (18). Studies demonstrate that cattle alter activity patterns in response to pain; decreased lying time is observed in cases of clinical mastitis due to a painful udder, whereas lameness is often associated with an increase in lying time to avoid applying pressure to a painful hoof (41). Thus, heightened tissue sensitivity leads to behavioral changes that promote healing by minimizing the risk of additional tissue damage. However, prolonged peripheral sensitization due to unrelieved pain can lead to long-term potentiation of neuronal synapses and disinhibition in the process known as central sensitization (42). Central sensitization may enhance neuronal excitability and diminish endogenous pain modulatory systems, leading to exaggerated nociceptor transmission and pain amplification (18). These alterations in the functional properties of neurons persist in the absence of the initiating insult (42), resulting in maladaptive pain. Studies in cattle suffering from chronic lameness demonstrate the development of central sensitization (43–46), leading to pain that is more refractory to treatment. Aversive contextual experiences, such as negative preslaughter conditions, may also predispose cattle to sensitization and heightened pain responses (30).

### 2.3 Neonatal neuroplasticity

From a young age, calves raised for dairy and beef production are subjected to painful procedures, and neonates may be particularly susceptible to the development of central sensitization and maladaptive pain. Research in human and rodent models suggests that the immature nervous system of neonatal animals is sensitive to somatosensory and nociceptive alterations in response to painful experiences (47). Rodent models demonstrate that neonatal tissue injury alters pain sensitivity; rats that experience injury early in development exhibit generalized hyposensitivity and localized hyperalgesia at the injury site in adulthood, as reviewed by Schwaller and Fitzgerald (48). This increased pain sensitivity at the injury site is likely related to alterations in neurocircuitry at the level of the dorsal horn of the spinal cord (48). Hyperinnervation resulting from the altered distribution of nociceptive fibers (49), microglial cell activation related to the developing immune system (50), and disruption to the descending modulatory system responsible for pain inhibition (47) have also been implicated in sensory disturbances arising from neonatal pain.

Evidence of altered tissue sensitivity in the aftermath of early life painful procedures has been observed in cattle. Calves that undergo both chemical and cautery disbudding may experience chronic sensitization that persists months beyond the post-procedural period (51–53). Although this pain is associated with protracted re-epithelialization, it may lead to central sensitization due to failure to provide analgesia at the onset of tissue trauma and throughout wound healing. Calves disbudded as neonates demonstrate enhanced tissue sensitivity in regions distal to the initial site of trauma (51) and an enhanced cardiac response to vaccine administration (54). Hypersensitivity associated with neuroma formation has been documented in heifers after tail docking (55), as well as other farm animals, including piglets (56) and lambs (57). Although neonatal pain and neuroplasticity in farm animals is understudied, human and rodent models suggest that these nociceptive alterations may persist into adulthood (47). Studies in rats further demonstrate that neonatal inflammation negatively impacts future female reproductive development (58), maternal care, and offspring stress resiliency (59), demonstrating intergenerational repercussions of early life pain. A study conducted by Clark et al. (60) provides evidence that ewes that undergo tail-docking early in life exhibit greater pain responses during parturition as adults, and ewes experimentally induced with an infection during the neonatal period give birth to offspring with reduced pain sensitivity.

## 3 Overview of livestock anesthesia and analgesia

Although pain control in farm animals is a considerable public concern, there is minimal legislation governing the use of analgesics. Surveys conducted in the U.S. regarding farm animal pain indicate that the general public supports higher welfare standards (61). The Ohio Survey of Food, Agricultural and Environmental Issues (62) reported that 75% of respondents agreed with the statement that “farm animals should be protected from feeling physical pain” and a more recent national online survey conducted by the Animal Welfare Institute (63) revealed that greater than 80% of consumers agreed that painful procedures should be performed with analgesia. This public position, however, has not translated into regulatory expansion regarding the use of analgesics in farm animals. Regulations currently exist for appropriate drug use, such as approved routes of administration and withdrawal intervals for food-producing animals; however, no regulations specifically enforce the use of analgesics to safeguard animal welfare. The American Veterinary Medical Association (AVMA) and the American Association of Bovine Practitioners (AABP) provide recommendations for the use of analgesia for some painful procedures, such as dehorning and castration (64–66). The AVMA acknowledges that these procedures “cause pain and discomfort” and advocates for “procedures and practices that reduce or eliminate these effects” (66). However, a 2021 survey study of U.S. veterinarians (*n* = 497), producer-veterinarians (*n* = 569), and producers (*n* = 121) conducted by Johnstone et al. (67) reported that, in calves less than 2 months old, 43.8% of respondents “never use” local anesthesia for surgical castration and 32.8% “never use” local anesthesia for dehorning. Industry efforts to encourage the use of anesthetics and analgesics are similarly limited. The Farmers Assuring Responsible Management (FARM) Program Version 4.0 standards require the use of pain relieving drugs for disbudding; however, the use of anesthetics and analgesics is not required for castration and branding, although these procedures are characterized as painful in the Animal Care Reference Manual (68). Furthermore, there is a deficiency of recommendations guiding the management of painful disease conditions.

Veterinary drugs are approved by national agencies, and their availability and associated withdrawal times vary widely between countries. In the U.S., the Food and Drug Administration (FDA) Center for Veterinary Medicine (CVM) approves animal drugs. The FDA CVM is an organization designed to secure human and animal health (69). Under the authority of the Federal Food, Drug, and Cosmetic Act (FFDCA), the FDA CVM publishes the Code of Federal Regulations (CFR), defining the regulations governing the introduction of veterinary drugs (69). Drug approval for commercial marketing requires (1) safety for the target species, human consumer, animal handler, and environment; (2) pharmaceutical efficacy for the stated purpose; (3) quality manufacturing; and (4) appropriate labeling, including safety, storage, handling, and withhold information (69). There is a scarcity of drugs labelled for pain control in cattle. Consequently, veterinarians must use medications to control pain in an extra-label manner under the Animal Medicinal Drug Use Clarification Act (AMDUCA) (70). To prescribe and administer extra-label drugs, a veterinarian must comply with AMDUCA regulations, including the establishment of a veterinary-client-patient relationship (VCPR) and strict adherence to withdrawal intervals to avoid violative residues (21). Kleinhenz et al. (71) describe this as a significant “regulatory burden” placed on veterinarians.

In a hospital setting, a multimodal approach to analgesia can be utilized to target transduction, transmission, modulation, and perception to prevent and treat pain in cattle within the confines of extra-label drug use. However, in field settings many of these available drugs are cost-prohibitive or cannot be safely dispensed due to legislation concerning controlled substances, such as the opioids. These limitations underscore the need for more FDA-approved analgesic options to facilitate safe, affordable, and effective methods of pain prevention and mitigation. Currently, the most common drugs used in livestock medicine include local anesthetics and nonsteroidal anti-inflammatory (NSAIDs) agents, although the use of these drugs remains inconsistent even for painful procedures with recommended guidelines (72). Refer to Table 1 for a detailed overview of available anesthetics and analgesics used in cattle medicine.

**Table 1:** Pharmaceutical options for pain prevention and mitigation in cattle.

Pharmaceutical options for cattle pain prevention & mitigation in the U.S.										
Drug	Drug class	FDA approval for pain control	Mechanism of action	Label indication in the U.S.	Dosage	Route of administration	T_1/2_	Meat WDI	Milk WDI	Comment
Lidocaine hydrochloride (2%) injectable solution	Local anesthetic	FDA-approved	Inhibits the generation of action potentials by blocking voltage-gated sodium channels (73)	Epidural, nerve conduction, and infiltration anesthesia for cattle, horses, dogs, and cats	1.5 mg/kg Max 15 mL for epidural Max 20 mL for nerve block	SC	4.19 (range: 2.1–7.26) h (74)	1 day (epidural) and 4 days (infiltration) if used according to label	24 h (epidural) and 72 h (infiltration) if used according to label	Currently the only local anesthetic FDA-approved for use in cattle (75) Sodium bicarbonate as a buffer may reduce injection pain (76), enhance analgesia (77), and reduce pharmaceutical activity onset (78); however, it may also reduce analgesic duration (78) Magnesium sulfate may prolong anesthetic duration (79)
Flunixin meglumine injection	NSAID	Extra-label	Inhibits COX enzymes, which catalyze the production of prostaglandins that mediate the inflammatory response (80) The COX enzyme has two known isoforms: COX-1 and COX-2. COX-1 is constitutively expressed and plays a fundamental role in tissue homeostasis by catalyzing the production of prostaglandins involved in gastric mucosal protection and renal blood flow maintenance (80). Historically, NSAIDs inhibited both isoforms of the COX enzyme; however, newer drugs have been formulated to achieve COX-2 selectivity and thus avoid the unwanted effects of NSAID use, including gastrointestinal and renal toxicity (80).	Control of fever associated with bovine respiratory disease and acute bovine mastitis and control of fever and inflammation associated with endotoxemia	1.1–2.2 mg/kg	IV	3.14–8.12 h (81–84)	4 days if used according to label. Extra-label recommendations: 7 days (single dose)	36 h if used according to label Extra-label recommendations: 84 h (single dose)	Flunixin is the only NSAID in the U.S. FDA-approved for use in beef and dairy cattle (approved for IV use only)
Flunixin transdermal solution	NSAID	FDA-approved Only approved to alleviate the pain associated with interdigital phlegmon (foot rot)		Control of fever associated with bovine respiratory disease and acute bovine mastitis, and the control of pain associated with foot rot in beef cattle 2 months of age and older and dairy cattle	3.3 mg/kg	TDRM	6.42 h (range: 5.22–9.76) (85)	8 days if used according to the label	48 h if used according to the label	The first and only drug with a label claim of pain control for cattle in the U.S. Recently expanded for use in lactating dairy cows (86)
Carprofen	NSAID	Extra-label		Relief of pain and inflammation associated with osteoarthritis and for the control of postoperative pain associated with soft tissue and orthopedic surgeries in dogs	1.4 mg/kg	IV, SC	30.7 h (healthy cows); 43.0 h (mastitic cows) (IV) (87)	Extra-label recommendations: 21 days	Extra-label recommendations: 0 h	
Ketoprofen	NSAID	Extra-label		Alleviation of inflammation and pain associated with musculoskeletal disorders in horses, dogs, and cats	1.5 mg/kg	IV, IM	0.42 h (IV) (88)	Extra-label recommendations: 7 days	Extra-label recommendations: 24 h	Only approved formulation in the U.S. is formulated with tulathromycin (Draxxin KP, Zoetis)
Meloxicam	NSAID	Extra-label		Control of pain and inflammation associated with osteoarthritis in dogs	0.5–1 mg/kg	PO	27 (range: 19.97–43.29) h (89)	Extra-label recommendations: 21 days	Depends on a cow's stage of lactation (90)	Meloxicam is beneficial due to its relatively long duration of action, preferential inhibition of the COX-2 enzyme (91), high bioavailability (92), and cost-effectiveness
Phenylbutazone	NSAID	Extra-label*		Relief of inflammatory conditions associated with the musculoskeletal system in horses and dogs	4 mg/kg	IV ONLY	40–55 h (93–95)	Extra-label recommendations: 55 days	NA	*Illegal for use in dairy cattle ≥ 20 months of age and its use is discouraged due to zero tolerance for residues
Firocoxib	NSAID	Extra-label		Control of pain and inflammation associated with osteoarthritis in dogs and horses and the control of postoperative pain and inflammation associated with soft-tissue and orthopedic surgery in dogs	0.5 mg/kg	PO	4.6–9.7 h (IV); 14.2–25.5 h (PO) (96, 97)	Extra-label recommendations: 96 days	Extra-label recommendations: 96 days Not recommended due to the lack of pharmacokinetic data in any species	COX-2 selective
Aspirin (acetylsalicylic acid)	NSAID	Extra-label		To reduce fever and for mild analgesia in beef cattle, dairy cattle, horses, calves and foals	50–100 mg/kg	PO	0.5 h (98)	1 day (no established WDI)	24 h (no established WDI); new data suggests that this WDI should be revised to 120 h (99)	Not formally approved by the FDA and cannot be used in lactating dairy cows Low oral bioavailability (21)
Xylazine	Alpha_2_-adrenergic agonist (Sedative analgesic)	Extra-label	Decreases norepinephrine release from presynaptic nerves, resulting in dose-dependent sedation and analgesia (21)	To produce a state of sedation accompanied by a shorter period of analgesia in horses and cervids	0.05–0.3 mg/kg (IM) 0.016–0.1 mg/kg (IV)	IV, IM	96.40 min when co-administered with ketamine and butorphanol IM (100)	Extra-label recommendations: 4 days (IM); 5 days (IV)	Extra-label recommendations: 24 h (IM); 72 h (IV)	The drug combination butorphanol (0.01 mg/kg)-xylazine (0.02 mg/kg)- ketamine (0.05–0.1 mg/kg), IV (“ketamine stun”) can be administered to enhance analgesia for procedural and surgical pain. This combination can also be administered IM or SC (0.015–0.02 mg/kg, 0.03–0.04 mg/kg, and 0.06–0.08 mg/kg) (101) Opioids are stringently regulated by the Drug Enforcement Administration (DEA). They cannot be dispensed on-farm
Ketamine	NMDA receptor antagonist (Dissociative anesthetic)	Extra-label	Impedes the activity of glutamate and binds to µ- and *κ*-opioid receptors, resulting in analgesia (102)	For use in cats for restraint or as the sole anesthetic agent for diagnostic or minor, brief surgical procedures. It may be used in non-human primates for restraint	5 mg/kg	IV, IM	67.43 min when co-administered with xylazine and butorphanol IM (100)	Extra-label recommendations: 3 days	Extra-label recommendations: 48 h	
Butorphanol	Synthetic opioid	Extra-label	Binds to opiate receptors, mimicking the activity of endogenous opioids (20)	For use in humans as a preoperative or pre-anesthetic medication, a supplement to balanced anesthesia, the relief of pain during labor, and the management of pain for which alternative treatments are inadequate	0.25 mg/kg	IM	68.23 min when co-administered with xylazine and ketamine IM (100)	Extra-label recommendations: 5 days	Extra-label recommendations: 72 h	
Gabapentin	GABA analogue (Anti-convulsant)	Extra-label	Inhibits the entrance of calcium into the nerve terminal, preventing the release of neurotransmitters (80)	Indicated for use in humans for postherpetic neuralgia in adults and adjunctive therapy in the treatment of partial onset seizures in adult and pediatric patients	10–20 mg/kg	PO	11.02 (range: 7.9–17.7) h at 10 mg/kg PO and 8.12 (range: 6.9–12.4) h at 15 mg/kg co-administered with meloxicam (0.5 mg/kg PO) (103)	Extra-label recommendations: 21 days	Extra-label recommendations: 3 days	Originally used as an anti-epileptic drug. Gabapentin may act synergistically with NSAIDS such as meloxicam, increasing therapeutic efficacy (103) May be useful for the treatment of chronic neuropathic pain associated with lameness in cattle (21)

Withdrawal interval recommendations are from the Food Animal Residue Avoidance Databank (FARAD). 2022. http://www.farad.org/wdilookup/wdi_cattle.html. The prescribing veterinarian must establish withdrawal intervals for drugs used in an extra-label manner for any food producing animal. Withdrawal intervals are based on drug dosage, as well as frequency and route of administration. They may also vary based on the age of the animal, stage of lactation, and disease status. Values in this table are subject to change as new pharmacokinetic data becomes available, and FARAD must be consulted for the most up-to-date recommendations. Drug dosages depend on frequency of administration and duration of treatment and should be determined by the established veterinarian.

WDI, withdrawal interval; T_1/2_, drug elimination half-life; IM, intramuscularly; IV, intravenously; SC, subcutaneously; PO (per os), orally; TDRM, transdermally; CRI, constant rate infusion, NMDA, N-methyl-D-aspartic acid.

### 3.1 Barriers to livestock anesthetic and analgesic use

There are numerous barriers to the use of analgesics in cattle. The scarcity of drugs labelled for pain control and consequent extra-label drug use may discourage producers from implementing pain management protocols due to the requirement for veterinary oversight under AMDUCA (104). Many of the drugs used in an extra-label manner must be administered intravenously and may require repeat doses, further inconveniencing producers, as animal restraint and a degree of skill are needed for this route of administration (21). The animal restraint involved in administration increases labor and may pose a risk to worker safety. The intervening time between analgesic administration and pharmaceutical activity may further discourage pain mitigation efforts by producers, who often have time constraints and many animals to process (21). These inconveniences are compounded by economic considerations, such as high cost and associated milk and meat withdrawal intervals (105). There is also a lack of studies investigating the economic benefits of pain management. Pain is associated with stress, depression, inappetence, and impaired wound healing and immune function (75)—all of which may negatively impact productivity. Studies that demonstrate an association between pain prevention and mitigation and increased animal productivity and longevity may incentivize producers to administer analgesics.

Pain detection and mitigation require shifts in attitude and effective veterinarian-producer communication. In a recent nationwide U.S. survey study of producers and veterinarians, Edwards-Callaway et al. (106) reported a significant association between respondent pain perception and analgesic administration; this is in accordance with previous studies conducted in the UK (107, 108). However, Remnant et al. (107) found that painful management procedures in young calves did not align with this trend. Although respondents recognized the pain associated with procedures such as disbudding and castration, they were less likely to provide postprocedural pain relief. The researchers propose several explanations for producer analgesic hesitancy: firstly, terminology such as “anti-steroidal” and “anti-inflammatory,” in contrast to simpler terms such as “pain killer,” may confuse a producer's understanding of the drug's purpose; secondly, the movement for antibiotic stewardship may encourage producers to avoid all injectable drugs, as they may conflate antimicrobial agents with anti-inflammatory agents. The misconception that neonatal animals do not experience pain may also contribute to inconsistency in analgesic use for these procedures (106). Veterinarians demonstrate a comparatively higher sensitivity to cattle pain than producers (106). Sumner et al. (109) assert that veterinarians, due to their training in pain management and working relationship with clients, are uniquely positioned to challenge the status quo and direct producers towards pain prevention and mitigation protocols.

Inconsistency in the use of pain relief for cattle can lastly be attributed to the difficulty of reliably assessing pain, as pain identification is fundamental to mitigation and required for drug approval as outlined by the CFR (69). A recent systematic review and meta-analysis conducted by Tschoner et al. (110) compared veterinarian and producer pain ratings for a variety of management procedures and disease conditions using the Numerical Rating Scale (NRS) and Visual Analogue Scale (VAS). The researchers concluded that inter-study comparisons were confounded by variations among pain scales, inconsistent terminology, and scorer variables, such as gender, age, and education, that contribute to the subjectivity of pain assessment (110). This underscores the need for sufficiently objective, reliable, and validated pain scales to guide on-farm pain detection.

## 4 Pain evaluation in cattle

Identifying reliable pain indicators enables us to determine pain intensity and analgesic efficacy, which are required for appropriate therapeutic intervention. Validation of pain indicators, however, is complicated by interspecies differences in the expression of pain. The available research on pain is primarily derived from human subjects with a reliance on self-report (111). Not only are non-human animals incapable of self-report, but findings from one species cannot be reliably extrapolated to another species due to differences in evolutionary history, selective pressures, and learned behaviors, which impact the observable expression of pain (112).

To survive predation, cattle evolved to mask pain. Disguising weakness and focusing on escape are important survival strategies with adaptive benefits (105). In contrast to other social animals, such as primates, dogs, and pigs, that vocalize to signal distress, vocalization may be maladaptive for a cow by signaling vulnerability to attack (112). Although the modern dairy farm does not mirror the predator-prey dynamic of the wild, there remain situations in commercial settings in which a cow benefits from disguising pain. For example, a dominant herd member may impose limits on feed access upon recognition of weakness in another cow (113). A dairy cow's consequent stoicism can obscure clinical pain identification and lead to the perception that cattle lack pain sensitivity (105). This highlights the importance of developing species-specific pain indicators; however, their validation is further complicated by intraspecies differences in pain expression.

The perception of pain—a subjective emotional experience—is revised by memories of prior experiences with noxious stimuli (114). For example, when pain is associated with a more pleasing environment, the pain intensity may be reduced; conversely, association with a negative environment may increase the pain response (18) (Section 2.1). An animal's degree of vigilance and affective state may impact the experience of pain (1) (Section 2.1), as can their developmental stage and current environment (105). Stress and reproductive factors, such as the estrous cycle, may also confound pain evaluation by altering behavioral and physiological parameters (105). Studies in mammals suggest that genetic differences may contribute to intraspecies variability in the pain response, as evidenced by the identification of more than 300 possible “pain genes” (115). Differences in the type and location of pain further account for variations in observable behavioral responses to pain among individual animals of the same species. Acute pain may present differently than chronic pain; superficial pain may present differently than visceral pain (105). An animal's breed and sex further account for intraspecies differences in pain detection. A study conducted by Martin et al. reported differences in behavioral and physiological indicators of pain between male and female Angus and Hereford calves in response to hot-iron branding (116).

Large scale farming additionally challenges pain detection; herd size within the U.S. dairy sector has increased dramatically with the average number of cows per herd reaching 1,300 in 2017 (117). Without a concomitant increase in worker number or implementation of automated monitoring systems, individual animal observation is compromised. Precision Livestock Farming (PLF) emerged in 2003 (118) and employs technology and machine learning to continuously monitor animals at both the individual and population levels in real-time, enabling the collection of large amounts of data while minimizing associated labor (119). PLF technologies may aid in the advancement of animal welfare, including pain detection and mitigation, by streamlining the detection of parameters associated with positive or negative welfare states. There are concerns that automated systems may encourage further agricultural intensification and negatively impact the human-animal bond (120); however, PLF technologies have the potential to enhance animal welfare if thoughtfully integrated into farm management systems and research initiatives (121). Although PLF technologies have not yet been widely adopted for pain detection, they have the potential to facilitate pain surveillance on large-scale operations. Furthermore, welfare and production parameters associated with pain can be studied in parallel to incentivize higher welfare practices.

Even when aided by automated systems, effective pain detection requires validated pain response measures that are reproducible between studies with maximized sensitivity and specificity. According to Weary et al. (4), the gold standard for validation requires studies that evaluate pain responses in the presence and absence of a painful condition and in the presence and absence of an analgesic drug known to effectively mitigate the pain caused by this condition. Despite the associated challenges, pain assessment can be guided by three categories of pain response: production, physiological, and behavioral indicators (4).

### 4.1 Evaluating pain in cattle: production indicators

Cattle production indicators include feed intake, average daily gain, milk yield, and fertility measures. Studies investigating performance outcomes are lacking in the available literature, as reviewed by Newton and O'Connor (122). Several studies have examined the influence of disbudding and castration on average daily gain and feed intake with mixed results (123–126), which Newton and O'Connor attribute, in part, to insufficient study periods and small sample sizes. Sufficiently robust studies are similarly lacking for the impact of disease-associated pain on production parameters. Lameness results in herd economic losses due to reduced milk yield (127–129) and impaired reproductive performance (130). Lameness also alters behaviors critical to production performance such as feed intake (131). Clinical metritis has been reported to result in reduced milk yield (132), impaired reproductive performance (133), and premature culling (134). However, these production parameters have not been examined in relation to the specific contribution of pain associated with the disease or management procedure.

Production performance indicators are not as useful for identifying immediate pain to guide treatment, though they may indicate chronic pain. Reduction in average daily gain or body condition score due to decreased feed intake, for example, may suggest protracted pain (4). However, changes in appetite may take several hours to detect, and days are required to detect loss of body condition (4). Thus, this measurement is not necessarily reflective of an animal's current experience. In addition to changes in feeding behavior, pain may increase stress levels and activate the immune system, altering nutrient utilization and negatively impacting body condition (135). Although production performance parameters do not capture the inner experience of the animal and are less important from the animal's point of view, they remain an important consideration, as economic incentive often drives pain mitigation efforts. Further research investigating the impact of pain on production performance is needed, as improved production may offset the cost of analgesics, incentivizing their use.

### 4.2 Evaluating pain in cattle: physiological indicators

**Figure 3:**
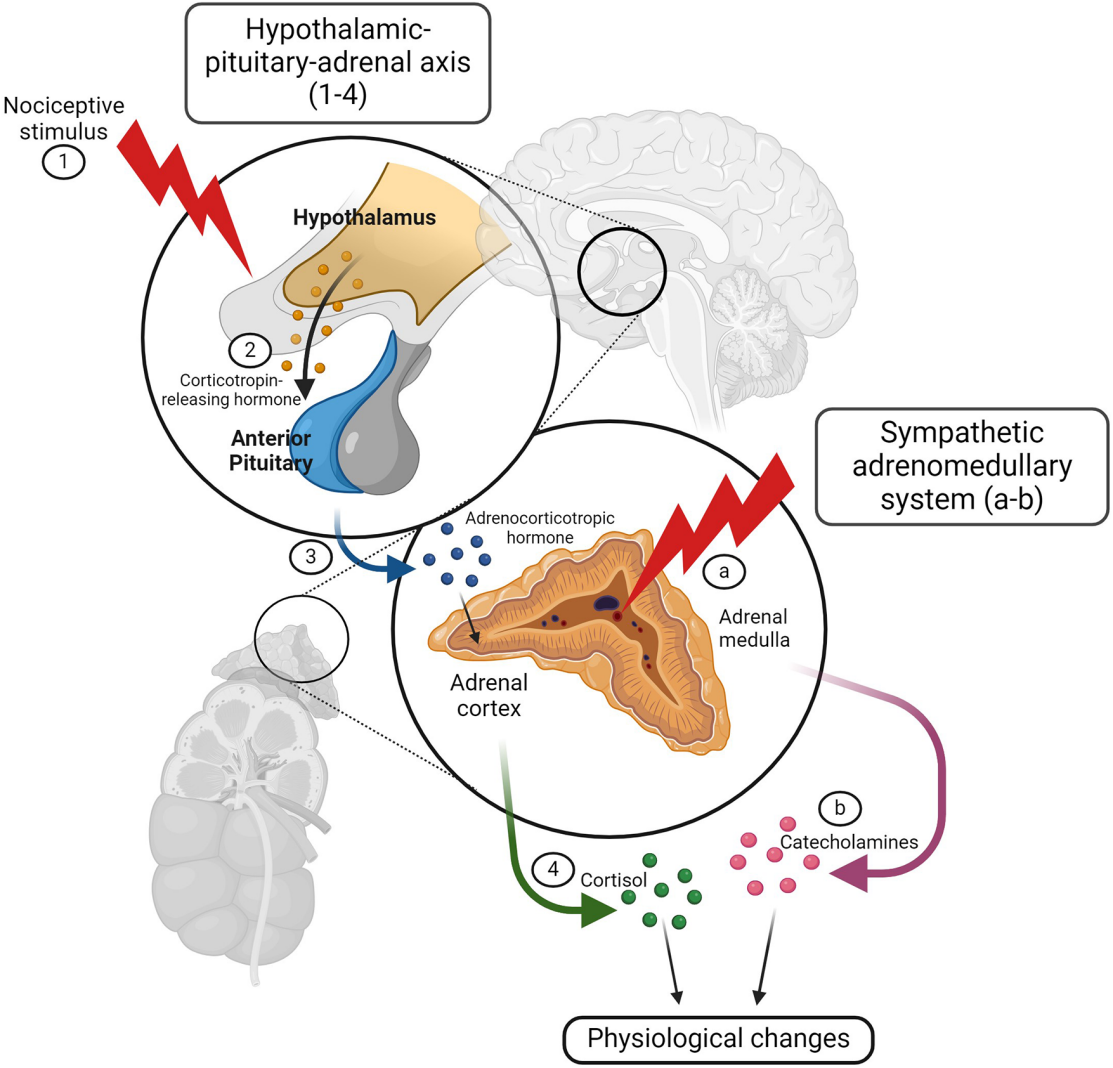
The hypothalamic-pituitary-adrenal axis and autonomic nervous system respond to noxious stimuli resulting in physiologic changes. A noxious stimulus activates the hypothalamic-pituitary-adrenal (HPA) axis and the sympathetic division of the autonomic nervous system (ANS). After processing the nociceptive signal (1), the hypothalamus releases corticotropin-releasing hormone (CRH) to the anterior pituitary (2), which then releases adrenocorticotropic hormone (ACTH) to the adrenal gland (3). Once stimulated, the adrenal cortex releases cortisol into the bloodstream (4), resulting in physiologic changes associated with the stress response. Nociception also stimulates sympathetic nerve fibers innervating the adrenal gland (**A**) causing catecholamine release from the adrenal medulla (**B**) Catecholamines, such as epinephrine and norepinephrine, are responsible for the “fight or flight” response. Figure adapted from (138). Created with BioRender.com.

Physiological measures of pain include cardiovascular, respiratory, endocrine, neurophysiological, electrophysiological, and immune responses (136). Nociception may result in physiological stress responses. Nociceptive signals are detected by the hypothalamic-pituitary-adrenal (HPA) axis and the sympathetic division of the autonomic nervous system (ANS) (135). The hypothalamus processes nociceptive signals and releases corticotropin-releasing hormone (CRH) to the anterior pituitary, which in turn releases adrenocorticotropic hormone (ACTH) to the adrenal gland. Once stimulated, the adrenal gland releases cortisol into the bloodstream (137) (Figure 3). Elevated cortisol levels have been detected in calves in response to disbudding (139), castration (140), and branding (141). Cortisol elevations have also been detected in cattle with mastitis (9, 142, 143) and lameness (144, 145). Acute nociception also stimulates sympathetic nerve fibers innervating the adrenal gland, resulting in catecholamine release from the adrenal medulla (146). This release of catecholamines, such as epinephrine and norepinephrine, results in vasoconstriction that directs blood centrally for the “fight or flight” response, increasing cardiac output and respiratory rate (140) (Figure 3). Flight behavior is commonly exhibited by cattle during painful procedures, such as hot-iron branding (147) and elevations in norepinephrine have been reported in lame cattle (148). Many studies examine heart rate, heart rate variability, and respiratory rate as indicators of pain in calves undergoing painful procedures (149) and cattle with lameness (150), mastitis (142), and metritis (13). The autonomic nervous system response to pain can also be captured with infrared thermography (IRT), which detects alterations in temperature distribution related to blood flow, demonstrating sympathetic nervous system activation. Using IRT, ocular temperature changes have been reported in calves undergoing disbudding (151, 152) and castration (27, 140, 149).

The immune response to tissue damage also provides opportunities for physiological measurements indicative of pain. Tissue damage leads to the release of inflammatory mediators involved in nociception, such as interleukin-1 (IL-1), a proinflammatory cytokine that stimulates the adrenal gland to release ACTH (153). Other inflammatory products, such as fibrinogen and haptoglobin, indirectly suggest pain presence, as the inflammatory process often evokes a state of pain (135). Elevated concentrations of haptoglobin have been reported in cattle with mastitis (9, 142, 143), metritis (154, 155), and lameness (150, 156, 157).

The neurotransmitter substance P has been studied as a pain biomarker in adult cattle and calves as reviewed extensively by Tschoner and Feist (158). Studies have investigated substance P concentrations in cattle suffering from lameness (144, 145, 156), metritis (154), and dystocia (159). Substance P has also been used to evaluate pain in calves that undergo castration (160) and dehorning (161).

Although physiological and neuroendocrine parameters can be easily quantified, their measurements frequently require specialized equipment and advanced skill sets (105). Studies are often more invasive than those analyzing behavioral indicators alone; measurements may require sample collection or fitting the animal with monitoring equipment (135). Physiological changes can also be caused by factors other than pain, such as stress and physical activity (105), minimizing testing specificity. This is exacerbated by collection techniques that may require animal restraint, thus increasing the likelihood that stress may confound data due to sympathetic nervous system activation (162). Lastly, the biological stress response indicates nociceptive pathway activation, which may not be accompanied by conscious pain perception. Animals under anesthesia, for example, demonstrate a physiological response to noxious stimuli and tissue damage in the absence of conscious perception (136). EEG signal variations (Section 2.1) and advanced imaging, such as positron emission tomography (PET) and functional magnetic resonance imaging (fMRI), have been used in human and animal models to provide evidence of central nervous system processing and perception of pain (163); however, these costly techniques remain impractical for farm animal pain assessment. Consequently, physiological parameters are best utilized collectively with parameters that more closely capture the inner experience of the animal, such as behavioral indicators.

### 4.3 Evaluating pain in cattle: behavioral indicators

Pain-specific behaviors include defensive and avoidance behaviors. When animals encounter a painful stimulus, they demonstrate a “nocifensive withdrawal reflex” (14). An animal may demonstrate an exaggerated withdrawal reflex to an innocuous stimulus due to hyperalgesia of an injured area or may alter body position to avoid stimulating a painful region (4). For example, a cow with mastitis may demonstrate hyperreactivity to udder contact during milking (143) and reduced lying times to avoid pressure applied to a painful quarter (164). To quantify escape behavior evoked by a painful procedure, researchers measure exertion force on a squeeze chute using load cells and strain gauges (147, 165); steers demonstrate greater exertion forces in response to hot iron branding when compared to freeze branding, providing insight into the relative pain associated with different methods of cattle identification (165). Animals may also engage in behaviors directed towards an affected area, such as licking and scratching (135). Calves were observed licking the scrotal region post-castration (166) and scratching their heads following disbudding (167). Head shaking and ear flicking are also used as behavioral indicators of pain in calves post-disbudding (168–170).

Quantitative sensory testing (QST) is a method used to obtain nociceptive threshold measurements based on a behavioral response to determine tissue sensitivity. QST techniques involve the application of a noxious stimulus to a specific body region to evoke a defensive behavior, such as withdrawal (136). Methods of eliciting a reaction include thermal sensitivity tests and mechanical nociception tests, such as pressure algometry (61). The resulting nociceptive threshold measurements, or the lowest magnitude of the applied stimulus needed to induce a response, can be used to determine tissue sensitivity (136). Nociceptor threshold measurements have been used to investigate tissue sensitivity in calves after painful procedures such as disbudding (51, 171–175) and castration (176, 177). They have also been used to evaluate tissue sensitivity associated with painful disease conditions such as mastitis (178), lameness (179–181), and respiratory disease in calves (182).

The goal of a study conducted by Ohlheiser et al. (183) was to create an experimental pain induction model in dairy cows that could be adopted by future researchers to validate pain response measurements and evaluate analgesic efficacy. The researchers experimentally induced pain in cows using an intramuscular injection of oxytetracycline. They measured the mechanical nociceptive thresholds in locations that received the pain inducing drug and those that received a sham injection. They then measured these thresholds in the same locations in treatment animals administered flunixin meglumine (2.2 mg/kg IV) and control animals administered saline. Although this experiment aligned with the gold standard approach of pain assessment in both the presence and absence of analgesia, it is important to assess pharmaceutical efficacy in the presence of pain caused by the targeted disease condition. Furthermore, this study evaluated only mechanical nociceptive thresholds. Assessing multiple pain responses can increase the sensitivity and specificity of evaluation methods (135), which is essential to determining appropriate analgesic intervention.

Changes in vocalization patterns, social interactions, feed consumption and rumination, grooming behavior, and physical activity may be associated with pain (135). Pedometers, accelerometers, and rumen telemetry facilitate data collection of activity pattern alterations due to illness and pain (184). A literature review conducted by Mainau et al. (41) evaluated the available studies on activity pattern variations in dairy cattle with painful disease conditions, such as mastitis, lameness, metritis, and dystocia.

Changes in posture and facial expression may also indicate experienced pain. Postural changes represent an attempt to mitigate discomfort and have been detected in cows with metritis (13), lameness (185, 186), and disorders of the gastrointestinal tract (187). Facial expression studies have gained recent traction with scales developed for farm animals, including sheep, pigs, and cows (188–192). In a study conducted by Gleerup et al. (192), researchers evaluated pain in dairy cattle using a variety of pain indicators, including facial expression. The “Cow Pain Face” includes tense or low ears, tense or withdrawn eyes, dilated nostrils, and muscle tension above the eyes, nostrils, and along either side of the head (192). Although the researchers reported higher scores in cows with a painful clinical diagnosis, additional studies are needed to evaluate the diagnostic ability of this scoring system. A study conducted by Yamada et al. (193) used facial expression to evaluate the pain associated with different castration methods in bulls. Automated systems that analyze facial expressions have been developed for sheep to enhance objectivity and avoid inter-observer variability (194).

Behavioral parameters have also been used to develop pain scales. Validated pain scales exist for horses (195), dogs (196–199), and cats (200, 201); however, there is a lack of validated pain scales developed specifically for cattle. The UNESP-Botucatu unidimensional composite pain scale was refined and validated in 2014 to evaluate postoperative pain in cattle that undergo an orchiectomy (202). With this scale, readily observable behaviors, such as activity level, eating frequency, posture, and behaviors directed towards the source of pain, provide a non-invasive method of assessment to guide analgesic intervention.

A primary advantage of behavioral studies is that they can be conducted on-farm with direct observation and minimal subject manipulation. Additionally, pain-specific behaviors can be used to localize a painful region, guiding targeted therapy (135). Behavioral indicators are also rapidly observable and sensitive, as changes in behavior occur as cattle experience pain in real time, enabling early detection for timely analgesic intervention (135). This sensitivity, however, is compromised by the subjective nature of behavioral observations, which rely on individual interpretation (203). Studies that evaluate observer identification of cattle pain indicate variability based on education, age, and gender (107, 110). To increase testing sensitivity, observers must be experienced and trained in bias avoidance. Testing specificity is undermined by alterations in behavior that may be due to sickness, stress, or discomfort rather than nociception (135). Lastly, as previously discussed, a cow's evolutionary predisposition to stoicism challenges pain identification based on behavioral indicators (4). However, behavioral studies bring us closer to the inner experience of the animal.

### 4.4 Evaluating pain in cattle: affective state

Although many physiological parameters, such as cortisol and heart rate, reflect emotional arousal, few approaches to pain evaluation capture emotional valence, or whether an emotional experience is positive or negative (204). Although behavioral measures may bring us closer to the inner experience of an animal, many of these measurements simply indicate nociceptive activation and tissue damage avoidance without providing insight into the animal's psychology. Emerging studies in animal cognition, however, promise access to this inner experience that is difficult to quantify. Cognitive bias tests attempt to illuminate emotional valence by evaluating an animal's response to an ambiguous stimulus. Anticipation of a negative outcome demonstrates a “pessimistic” or negative affective state (136). Studies demonstrate a negative cognitive bias in calves following hot iron disbudding (205, 206), providing evidence that pain impacts emotional state. In a study conducted by Neave et al. (205), calves were trained to nose-touch a red and white screen; activation of the red screen resulted in a milk reward, whereas activation of the white screen resulted in a milk “time out.” Researchers found that within the 22 h following cautery disbudding, calves were less likely to approach ambiguously colored screens, indicating a negative cognitive bias. Reduction in play behavior (207–209) and intake of a sweetened solution (210) post-disbudding suggest that calves experience anhedonia—or disinterest in activities they once found pleasurable—as a result of experienced pain. Gingerich et al. (211) reported calves exhibiting “social withdrawal” following disbudding, as evidenced by a greater tendency to retreat to a secluded, sheltered area after this painful event. Anhedonia is associated with depression and anxiety in humans (212) and may include social isolation (213).

Conditioned place preference and avoidance tests further seek to illustrate an animal's perception of pain by demonstrating that, when provided the choice, animals will engage in behaviors that minimize the risk of repeated encounters with a painful experience. This was demonstrated by Ede et al. (214) in which calves chose to spend less time in the disbudding pen than the control pen (214). Providing animals with the agency to mitigate pain is demonstrated by self-medication trials in which animals are presented with the opportunity to consume feed containing analgesics. Danbury et al. (215) demonstrated that lame broiler chickens consumed more treated feed compared to control animals, and the degree of consumption correlated with lameness severity. Adcock and Tucker (168) studied disbudded versus control calves' motivation to obtain analgesia by pairing different visual stimuli with either a lidocaine injection or a saline injection. The researchers found that, 3 weeks after disbudding, the control calves demonstrated conditioned place aversion to the lidocaine-paired stimulus, whereas disbudded calves did not avoid the lidocaine-paired stimulus. The researchers concluded that disbudded calves were willing to absorb the aversive costs associated with a lidocaine injection to obtain relief for the more persistent pain resulting from disbudding. A study conducted by Colston et al. (216) provides evidence that calves are motivated to obtain cold therapy post-disbudding. Prior to disbudding, researchers exposed calves to a milk reward paired with habituation to cold and ambient packs applied to their horn buds. Following disbudding, calves more quickly approached and remained in contact with the milk reward associated with cold pack application, demonstrating motivation to obtain cold therapy for pain relief. According to Sneddon et al., to demonstrate the experience of pain rather than simply nociception, an animal must exhibit a “change in future behavioral decisions and motivational changes” (14). Tests that illuminate an animal's emotional state, memory-based avoidance behavior, and the decision to self-medicate thus provide evidence of the conscious perception of pain in animals.

## 5 Discussion: cattle pain and welfare

The fundamental mechanisms, nociceptive pathways, and central nervous system structures that lead to pain perception are highly conserved among mammalian species (217). We can therefore infer that cattle experience an aversion to pain based on commonalities in our underlying neuroanatomy and physiology. However, we must draw caution when relying on humans as a comparative model due to interspecies differences in pain expression—most notably a cow's inherent stoicism. Using an anthropomorphic paradigm to shape our understanding of pain expression can lead to the assumption that cattle experience a blunted pain response or fail to perceive pain altogether. Stoicism is a behavioral state deeply rooted in a cow's evolutionary history, and we must acknowledge this predisposition to conceal vulnerability when investigating appropriate measurements to identify pain and assess analgesic efficacy.

There is empirical evidence that cattle respond to pain as demonstrated by alterations in physiological, neuroendocrine, and behavioral parameters. These measurements bring us closer to understanding the experience of an animal in pain. Emerging studies in animal cognition promise greater access to an animal's inner experience. Studies that aim to assess preference and motivation, such as conditioned place aversion and cognitive bias tests, are gaining momentum. Available data illuminate the significant influence of pain on animal welfare as indicated by lasting memories of painful experiences (214), anhedonia (207, 210), and behaviors indicative of a negative affective state in animals following a pain-inducing event (205). Although these approaches require continued investigation and validation, available data reveal that, like humans, cattle experience psychological distress associated with pain.

### 5.1 Unabated pain, central sensitization, and cattle welfare

The impact of pain on welfare is further revealed by studies demonstrating the development of pathological pain states when pain is left untreated. Acute pain does not always dissipate with time; it may become chronic and pathologic when protracted. The mechanisms that cause and maintain central sensitization are complex; they encompass multiple molecular pathways and mechanisms, as well as a multitude of both neuronal and non-neuronal contributors. Although the causes and mechanisms of central sensitization have not been fully elucidated, current evidence revises historical notions that pain results only from pathology or noxious stimuli arising from the periphery. Pain can manifest from central neuronal plasticity due to prolonged and unabated pain, leading to fundamental functional alterations to the properties of neurons (218). These functional alterations may result in spontaneous nociceptor activity, reduced nociceptor thresholds, and expansion of nociceptor receptivity to both noxious and innocuous stimuli (19), amplifying pain. The available research provides evidence of chronic pain in cattle suffering from lameness (45, 219) and that prolonged lameness may lead to central nervous system alterations (43).

Further research investigating pain chronicity and the development of central sensitization in farm animals is needed to guide analgesic intervention and ensure adequate pain relief duration to prevent the development of pathological pain. There is a scarcity of studies evaluating neuropathic pain, and analgesic options for these conditions are severely limited. In human medicine, self-report of pain has enabled evidence-based treatment of neuropathic pain, which includes anticonvulsant, antidepressant, opioid, and cannabinoid pharmaceutical agents (220). There is minimal research investigating the use of these drugs in farm animals. Cross-disciplinary and transspecies engagement may advance pharmaceutical approaches to chronic pain management in cattle; however, novel pain relief investigation is hindered by the lack of established pain evaluation methods. Studies in humans demonstrate that neuropathic pain is associated with depression (221) and anxiety (222), and available research in cattle suggest similar negative emotional outcomes (Section 4.4), highlighting the need for additional research in this area to secure welfare.

### 5.2 Early life painful procedures and cattle welfare

Although it is important to perform painful management procedures at the “earliest age practicable” to minimize tissue damage (66), preemptive and multimodal analgesic approaches are still required early in life to safeguard welfare. Not only are these procedures painful in young animals, but the neonate may be particularly susceptible to long-term negative welfare outcomes due to the plasticity of the developing nervous system (48). When exposed to noxious stimuli, the immature nervous system may experience detrimental somatosensory and pain processing alterations (47). In addition to altered pain sensitivity, rodent models demonstrate that inflammation experienced in early life can have adverse effects on fear, anxiety, and stress responses, as well as cognitive and social development (223). More studies investigating farm animal neonatal neuroplasticity in the context of painful early life procedures are needed. Where research is lacking in these species, a comparative approach to understanding the negative long-term consequences of unrelieved pain using other animal models should be applied to prioritize pain management on farms.

The long-term welfare implications of neonatal pain underscore the need for alternative methods of management and genetic advances to preclude the need for painful procedures. This includes the use of sexed semen (224) and immunocastration (225) to minimize the need for castration, and the advancement of polled genetics (226) to eliminate the need for disbudding. When these procedures are required for animal and human safety and welfare, they must be performed as early as appropriate to minimize tissue damage, the least painful method should be employed, and a preemptive and multimodal analgesic approach implemented. Although the combined use of a local anesthetic and NSAID are considered standard practice for painful procedures such as disbudding due to veterinary and industry-driven guidelines (64, 65, 227), ensuring adequate duration analgesic relief based on pain chronicity is needed. Adcock and Tucker (51) reported that, following cautery disbudding, calves experience “evoked pain,” or increased sensitivity in response to a stimulus, for 9 weeks and “ongoing pain,” or pain experienced in the absence of a stimulus, for at least 3 weeks. This suggests that a single NSAID dose is insufficient in mitigating post-procedural pain following disbudding. It is also important to reduce situational stress through appropriate handling and restraint, as fear and anxiety may enhance the perception of pain (Section 2.1). Practical means of implementing positive reinforcement in farm settings should also be investigated, as pairing a painful stimulus with a reward may promote pain attenuation. However, we consider these methods of situational stress reduction supplemental and not as a replacement for appropriate analgesic intervention.

It is also important to note that the pain associated with disbudding and castration is well-documented in the literature; however, there are other understudied painful procedures that we must examine to enhance cattle welfare. Additional studies are needed to evaluate the pain associated with branding, ear tagging, and electroejaculation, as well as effective analgesic options to mitigate this pain. Although there are studies demonstrating that these procedures are painful (141, 228, 229), there is relatively less public awareness about them, minimizing efforts to prioritize alternative methods of management and analgesic intervention.

### 5.3 Painful disease conditions and cattle welfare

When approaching infectious disease treatment on farms, priority is given to disease resolution via the use of antimicrobial agents while the pain associated with disease receives less attention in clinical decision making. Although veterinary and industry recommendations exist to guide pain mitigation for painful procedures (64, 65, 227), similar guidelines are not widely available to guide the management of painful disease conditions. There is also minimal research investigating the pain associated with disease in cattle, especially diseases such as metritis and respiratory illness. Consequently, the pain associated with disease is frequently undertreated or untreated. Research that investigates the pain associated with cattle disease is needed to implement evidence-based analgesic protocols. Improved management practices are also required to reduce the risks associated with disease development. For example, we can minimize the risk of lameness through environmental management, scheduled hoof trimmings, and appropriate nutrition (230). Genetic selection for traits that prioritize health and resilience may also reduce the incidence of production-associated diseases.

The importance of pain prevention and mitigation applies to all animals raised for food (71). Although this review focuses on pain in cattle, we need to draw attention to pain in all species, especially those domesticated for human use. Similar challenges to analgesic protocol implementation exist among operations rearing different farm animal species; these challenges include constraints related to pain detection, attitudes towards an animal's experience of pain, insufficient pain management guidelines, and the scarcity of FDA-approved drugs labelled for pain control (71). The barriers to pain prevention and mitigation are not insurmountable; further research investigating and validating bovine indicators of pain, veterinarian-driven initiatives to communicate the importance of pain management, and public awareness of the detrimental impact of pain on cattle welfare together may shift society towards farm animal pain prioritization and incentivize drug manufacturers to pursue drug approval. The available literature indicates that we must implement preemptive and multimodal pain management protocols to address the welfare of animals under our care; however, pain management prioritization—even in the presence of regulatory requirements—hinges on shifts in attitude toward pain and available, convenient, and effective analgesic options.
